# Computerized Cognitive Behavioral Therapy for Treatment of Depression and Anxiety in Adolescents: Systematic Review and Meta-analysis

**DOI:** 10.2196/29842

**Published:** 2022-04-11

**Authors:** Alice Wickersham, Tamara Barack, Lauren Cross, Johnny Downs

**Affiliations:** 1 Institute of Psychiatry, Psychology & Neuroscience King's College London London United Kingdom; 2 Department of Psychiatry University of Cambridge Cambridge United Kingdom

**Keywords:** adolescent, anxiety, depression, meta-analysis

## Abstract

**Background:**

Depression and anxiety are major public health concerns among adolescents. Computerized cognitive behavioral therapy (cCBT) has emerged as a potential intervention, but its efficacy in adolescents remains unestablished.

**Objective:**

This review aimed to systematically review and meta-analyze findings on the efficacy of cCBT for the treatment of adolescent depression and anxiety.

**Methods:**

Embase, PsycINFO, and Ovid MEDLINE were systematically searched for randomized controlled trials in English, which investigated the efficacy of cCBT for reducing self-reported depression or anxiety in adolescents aged 11 to 19 years. Titles, abstracts, and full texts were screened for eligibility by 2 independent researchers (TB and LC). A random-effects meta-analysis was conducted to pool the effects of cCBT on depression and anxiety symptom scores compared with the control groups. Study quality was assessed using the Cochrane Collaboration Risk of Bias tool.

**Results:**

A total of 16 randomized controlled trials were eligible for inclusion in this review, of which 13 (81%) were included in the meta-analysis. The quality of the studies was mixed, with 5 (31%) studies rated as good overall, 2 (13%) rated as fair, and 9 (56%) rated as poor. Small but statistically significant effects of cCBT were detected, with cCBT conditions showing lower symptom scores at follow-up compared with control conditions for both anxiety (standardized mean difference −0.21, 95% CI −0.33 to −0.09; *I^2^*=36.2%) and depression (standardized mean difference −0.23, 95% CI −0.39 to −0.07; *I^2^*=59.5%). Secondary analyses suggested that cCBT may be comparable with alternative, active interventions (such as face-to-face therapy or treatment as usual).

**Conclusions:**

This meta-analysis reinforces the efficacy of cCBT for the treatment of anxiety and depression and is the first to examine this exclusively in adolescents. Future research could aim to identify the active components of these interventions toward optimizing their development and increasing the feasibility and acceptability of cCBT in this age group.

**Trial Registration:**

PROSPERO CRD42019141941; https://www.crd.york.ac.uk/prospero/display_record.php?RecordID=141941

## Introduction

### Background

Depression and anxiety represent significant public health concerns among adolescents [1]. Depression and anxiety in adolescence are associated with negative outcomes which can extend into adulthood, such as suicidal behavior, risk of mental health disorders, substance abuse, poorer educational attainment, and poorer social functioning [2-5]. Cognitive behavioral therapy (CBT), a collaborative model of therapy, is widely considered the gold standard psychological intervention for the treatment of adolescent depression and anxiety [6,7]. Several reviews and meta-analyses have found it to be an effective treatment method, with improvements maintained during long-term follow-up [8,9].

However, many young people do not receive the help they need [10]. There are barriers to accessing treatment at the service level such as a lack of resources, inadequate staff training, limited availability of age-specific treatments, and difficulties in liaising with families [11]. Barriers at the patient level may include perceived stigma, confidentiality concerns, inability to access services, and a preference for self-reliance [12].

Computerized CBT (cCBT) programs, which deliver CBT digitally, may overcome some of these barriers. As heavy users of technology [13], cCBT programs may present various advantages over traditional face-to-face therapies for adolescents, such as reduced costs, accessibility, convenience, flexibility, and the avoidance of stigma associated with accessing traditional mental health services [14,15]. These programs vary widely in design and access. Many combine different types of media such as text, pictures, videos, activities, and gamification to engage adolescent audiences; some also include homework activities, personalization, and chat functionalities with trained therapists or administrators [14,16].

Previous systematic reviews and meta-analyses of the efficacy of digitally delivered CBT for anxiety or depression in children and young people have yielded promising results [14,17-23]. However, these reviews have typically been insensitive to age-specific effects, making it difficult to determine whether cCBT is equally effective for children, adolescents, and young adults. Ebert et al [18] stratified their meta-analysis by age group and found that studies examining cCBT among adolescents achieved better outcomes than those targeting children or mixed age groups. However, they examined studies published over 6 years ago (much has changed in digital health since then), with few available studies focusing on adolescents. More recently, Grist et al [19] stratified their meta-analysis by age group but did not differentiate cCBT from other technological interventions in doing so. Given that the National Institute for Health and Care Excellence (NICE) guidelines for depression in children and young people have recently recommended the use of digital CBT for mild depression, a strong and cCBT-specific evidence base for these technologies is crucial [7].

### Objectives

The aim of this systematic review of randomized controlled trials (RCTs) is to update previous meta-analyses and focus exclusively on the adolescent age group to investigate the efficacy of cCBT for treating anxiety and depression. Adolescence represents a distinct developmental period of biological and social transition [24] during which the prevalence of mental health disorders increases [25,26]. Furthermore, adolescents have distinctive technology and internet use habits compared with other age groups [13,27]. Therefore, it is important that adolescence be investigated as a distinct developmental period to evaluate whether cCBT is an effective intervention in this age group.

## Methods

The study was conducted in accordance with the PRISMA (Preferred Reporting Items for Systematic Reviews and Meta-Analyses) guidelines (Table S1 in Multimedia Appendix 1) [28] and was registered on PROSPERO (CRD42019141941).

### Ethics Approval

This review makes use of already published data, so ethical approval was not required or sought.

### Eligibility Criteria

Studies were included if (1) participants were aged 11 to 19 years (inclusive); (2) they examined any digital, computerized, or web-based CBT program; (3) they included depression or anxiety-related primary outcome measures; (4) they were completed RCTs; and (5) they were in English and published in a peer-reviewed journal.

The age criterion ranged from 11 years, matching the typical starting age for secondary school (United Kingdom) or middle school (United States), to 19 years, matching the upper threshold for adolescence used by the World Health Organization [29]. The RCT criterion was assessed based on the NICE definition of RCT [30]. No specific restrictions were placed on how depression or anxiety was operationalized; for example, they could be measured using self- or informant-rated symptom questionnaires or diagnostic criteria. In addition, any anxiety subtype was eligible for inclusion. No restrictions were placed on how cCBT was used as an intervention in the trials, such that the included programs could be preventive or treatment focused.

### Search Strategy

Reviewer 1 (TB) conducted the database and reference list searches. PsycINFO, Embase, and Ovid MEDLINE were searched up to July 1, 2019. Keyword searches were grouped around three concepts: (1) age group, (2) cCBT interventions, and (3) depression or anxiety. Database filters were not applied (Table S2 in Multimedia Appendix 2). The reference lists of included articles and relevant existing systematic reviews were also searched for potentially relevant studies. Reviewers 1 (TB) and 2 (LC) independently conducted title, abstract, and full-text screening according to the study eligibility criteria. Disagreements were discussed until consensus was reached. Reviewers 1 (TB) and 2 (LC) fully agreed on the final papers to be included in the review.

### Data Extraction

Reviewer 1 (TB) extracted study characteristics from the included full texts, such as information on the participants (country, study population, exclusion criteria, sample size, and participant age and gender), cCBT intervention or interventions, and control group or groups. Reviewer 3 (AW) extracted data to be used in the meta-analysis, such as information on the outcomes (outcome measurement, means, SDs, and SEs) and study design (randomization type, analysis type, and follow-up sample sizes). Wherever possible, data were extracted from intention-to-treat analyses. If studies reported outcomes for multiple follow-up periods, data were extracted for the longest follow-up period for which outcomes were reported in all relevant study arms (Cochrane Handbook section 9.3.4 [31]). If studies reported data from multiple relevant depression or anxiety measures, we extracted the study’s primary outcome measure by default, unless one of the secondary outcome measures was more similar to those used by the other included studies or if the outcome measure showed strong evidence of skew (Cochrane Handbook section 9.4.5.3 [31]). Reviewer 2 (LC) independently extracted the study characteristics and meta-analysis data of 10% of the included studies. The extraction agreement was high (81%). Following recommendations arising from peer reviews, reviewer 3 (AW) further extracted information on whether each study assessed treatment versus prevention, the role of parents in each intervention, and contacted corresponding authors for information on whether the interventions were available or delivered outside of the research setting (eg, in schools or in routine clinical care).

### Study Quality

Study quality was assessed using the Cochrane Collaboration Risk of Bias tool [32]. This tool assessed seven areas for possible bias (scored as low risk, high risk, or unclear risk): random sequence generation, allocation concealment, blinding of participants and personnel, blinding of outcome assessment, incomplete outcome data, selective reporting, and other biases. The overall study quality score (good, fair, or poor) was then calculated using the thresholds for converting the Cochrane Collaboration tool to the Agency for Healthcare Research and Quality standards [32,33]. Reviewers 1 (TB) and 2 (LC) independently assessed the risk of bias. Disagreements were discussed until consensus was reached.

### Statistical Analysis

We conducted meta-analyses to pool differences in follow-up depression and anxiety scores between the treatment and control arms. All analyses were conducted using Stata (version 15.0; StataCorp) [34]. Owing to anticipated heterogeneity in the study designs and scales used, we specified a random-effects model to pool standardized mean differences (SMDs) between the treatment and control arms using the Hedges correction [35,36]. For the main meta-analysis, if studies reported on multiple relevant study arms, we combined them to ensure that a single average treatment score was compared with a single average control score (Cochrane Handbook section 16.5.4 [31]). Heterogeneity was investigated using Cochran *Q* and the *I^2^* statistic, and publication bias was investigated using funnel plots and the Egger test for funnel plot asymmetry [37,38].

We conducted post hoc sensitivity analyses to investigate the effects of pooling different intervention types (treatment vs prevention), pooling different randomization techniques (clustered vs other), and pooling different analysis types (intention to treat vs other or unclear). We also investigated the effects of study quality, stratifying analyses by the overall study quality score (good, fair, or poor). Finally, we conducted a post hoc sensitivity analysis to investigate whether the pooled effect size varied according to the type of control group under study: active treatment (specific interventions or treatment as usual) versus other (waitlist, attentional, and no intervention controls). For this analysis, we did not combine multiple control arms into a single average control score as described above, such that studies could appear twice if they included 2 control arms (both active treatment and others).

## Results

### Study Characteristics

**Figure 1:**
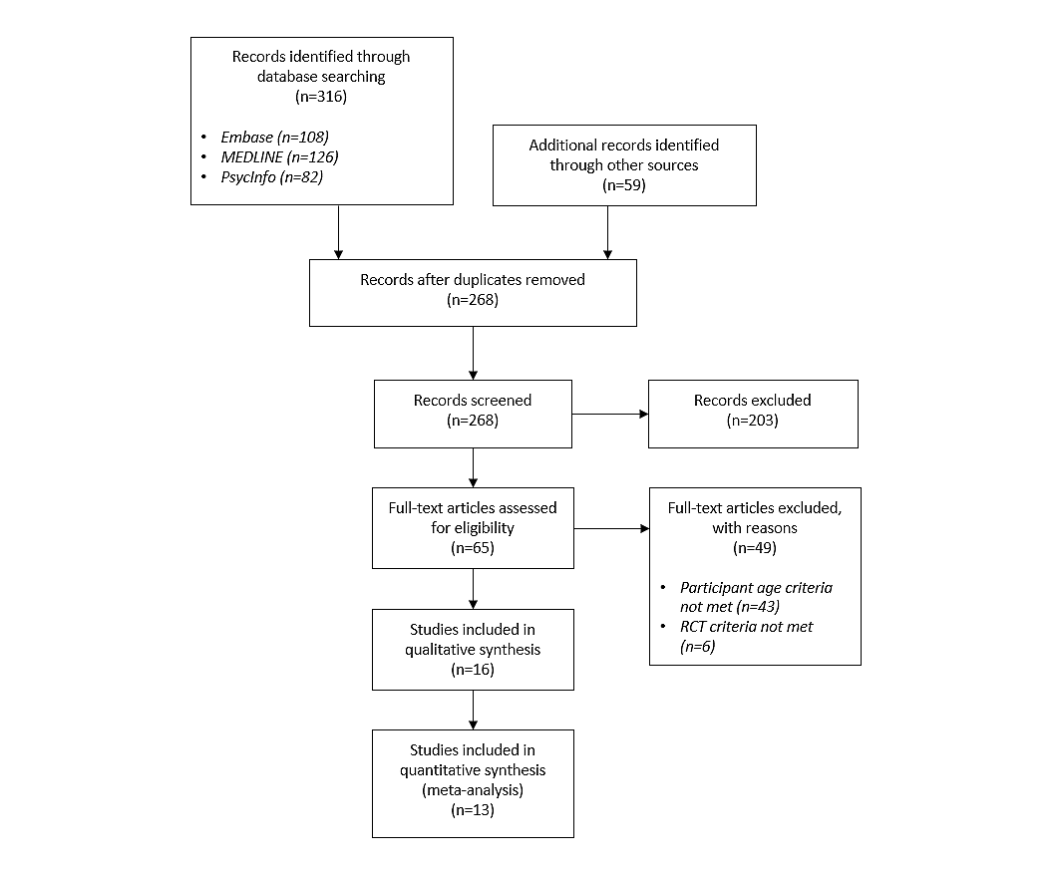
PRISMA (Preferred Reporting Item for Systematic Reviews and Meta-Analyses) flow diagram for study inclusion. RCT: randomized controlled trial.

A total of 16 studies were included in this review (Figure 1). They included 4012 participants, with all participants aged in the range of 11 to 19 years and with varying gender balances (Table 1). The studies targeted various populations (Table S3 in Multimedia Appendix 3 [39-54]) and were conducted in a wide range of countries, including New Zealand, China, Japan, Australia, the United Kingdom, the Netherlands, Denmark, and Sweden. Studies had a range of participant exclusion criteria, most often excluding participants who had severe symptoms, had other disorders, were at high risk of self-harm or suicide or were already receiving treatment (Table S3 in Multimedia Appendix 3 [39-54]).

Interventions were conducted at school, at the participant’s home, or in a setting of the participant’s choice, such as a local child and adolescent mental health service, local general practice, and community center. The extent of clinician or therapist input varied among interventions but was typically minimal. In addition, most interventions did not require parents to take an active role, with the exception of 19% (3/16) of the studies in which parents received additional guidance to support their adolescents through the course of treatment [39-41]. Some of the studies investigated interventions that have been made publicly available, whereas others have not been implemented beyond the research setting. A description of each cCBT program is provided in Multimedia Appendix 4 (Table S4) [39-54].

**Table 1:** Study characteristics.

Study	Country	Participants, n	Age (years), mean; range	Gender, male (%)	Intervention arm or arms	Control arm or arms
Calear et al [42]	Australia	1477	14.34; 12-17	44	MoodGYM	Waitlist
Fleming et al [43]	New Zealand	32	14.9; 13-16	56	SPARX^a^	Waitlist
Ip et al [44]	China	257	14.63; 13-17	32	Grasp the Opportunity (culturally modified from CATCH-IT^b^)	Attention control (antismoking website without mental health prevention components)
Merry et al [45]	New Zealand	187	15.6; 12-19	34	SPARX	Treatment as usual (face-to-face therapy)
Poppelaars et al [46]	Netherlands	208	13.35; 11-16	0	SPARX	School-based CBT^c^; monitoring control^d^
Sekizaki et al [47]	Japan	80	15.75; 15	100	Group iCBT^e^ program	No intervention
Smith et al [48]	United Kingdom	112	Not reported; 12-16	Not reported	Stressbusters	Waitlist
Spence et al [39]	Australia	115	13.98; 12-18	41	BRAVE-Online	Clinic-based CBT; waitlist
Sportel et al [49]	Netherlands	240	14.21; 12-15	28	Online Cognitive Bias Modification	Face-to-face CBT; no intervention
Stallard et al [50]	United Kingdom	20	Not reported; 11-17	67	Think, Feel, Do	Waitlist
Stasiak et al [51]	New Zealand	34	15.2; 13-18	59	The Journey	Attention control (computerized psychoeducation)
Stjerneklar et al [40]	Denmark	70	Not reported; 13-17	79	ChilledOut Online	Waitlist
Topooco et al [52]	Sweden	70	17.04; 15-19	Not reported	Blended approach with weekly therapist chats	Attention control (restricted access to platform and therapist chat but not the CBT component)
Wong et al [53]	Australia	976	Not reported; 14-16	30	ThisWayUp Schools (modules: “Overcoming Anxiety” or “Combating Depression”)	Usual health classes
Wright et al [54]	United Kingdom	91	15.34; 12-18	34	Stressbusters	Attention control (self-help website)
Wuthrich et al [41]	Australia	43	15.7; 14-17	37	Cool Teens CD-ROM	Waitlist

^a^SPARX: Smart, Positive, Active, Realistic, X-factor thoughts.

^b^CATCH-IT: Competent Adulthood Transition with Cognitive Behavioral Humanistic and Interpersonal Training.

^c^CBT: cognitive behavioral therapy.

^d^A fourth arm consisted of both SPARX and school-based CBT but was not analyzed as part of this review, as for our purposes it was a combined treatment and control group.

^e^iCBT: internet-based CBT.

### Study Quality

Study quality was mixed, with 31% (5/16) of the studies rated as good overall, 13% (2/16) rated as fair, and 56% (9/16) rated as poor (Table 2). There was a low risk of bias in 88% (14/16) of the included studies for the completeness of outcome data and other biases, 81% (13/16) for random sequence generation, and 56% (9/16) for allocation concealment and blinding of the participants and personnel. There was a low risk of bias among only 50% (8/16) of studies for the blinding of outcome assessment and 19% (3/16) of studies for selective reporting. The risk of bias in the remaining studies was typically unclear.

**Table 2:** Results of risk of bias assessment.

Study	Random sequence generation	Allocation concealment	Blinding of participants and personnel	Blinding of outcome assessment	Incomplete outcome data	Selective reporting	Other bias	Overall study quality
Calear et al [42]	+^a^	+	?^b^	?	+	?	+	Poor
Fleming et al [43]	+	+	+	+	+	+	?	Good
Ip et al [44]	+	+	+	+	+	?	+	Good
Merry et al [45]	+	+	+	+	−^c^	+	+	Fair
Poppelaars et al [46]	+	?	+	+	+	?	+	Poor
Sekizaki et al [47]	−	?	−	−	+	?	+	Poor
Smith et al [48]	+	?	?	?	+	?	+	Poor
Spence et al [39]	+	?	+	+	+	?	+	Fair
Sportel et al [49]	+	+	+	+	+	+	+	Good
Stallard et al [50]	?	+	?	?	+	?	+	Poor
Stasiak et al [51]	+	+	+	+	+	?	+	Good
Stjerneklar et al [40]	+	+	+	+	+	?	+	Good
Topooco et al [52]	+	+	+	−	+	?	+	Poor
Wong et al [53]	?	?	?	?	−	?	+	Poor
Wright et al [54]	+	?	?	?	+	?	+	Poor
Wuthrich et al [41]	+	?	?	?	+	?	−	Poor

^a^+: low risk of bias.

^b^?: unclear risk of bias.

^c^−: high risk of bias.

### Efficacy of cCBT

In all, 19% (3/16; 2 of poor quality and 1 of good quality) did not report adequate data to be included in the meta-analyses [43,47,50]. Here, we briefly summarized the findings of these studies. Compared with waitlist control groups, Sekizaki et al [47] found better depression and anxiety symptoms following cCBT, whereas Fleming et al [43] found this only for depression. Stallard et al [50] found symptom improvements in both cCBT and waitlist control groups but did not conduct sufficient analyses to assess whether either group had better outcomes than the other group. For the remaining studies, we present the meta-analysis results separately for anxiety and depression outcomes. The data used in the meta-analyses can be found in Multimedia Appendix 5 (Table S5) [39-42,44-46,48,49,51-54].

### Anxiety Meta-analysis

**Figure 2:**
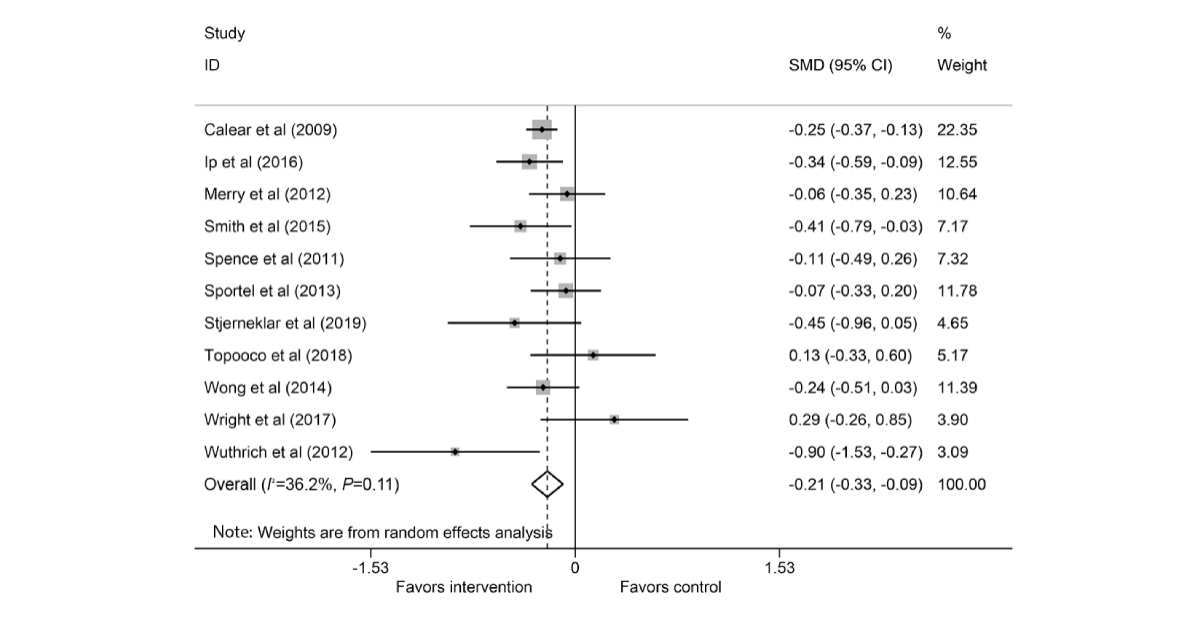
Forest plot for anxiety meta-analysis. SMD: standardized mean difference.

**Figure 3:**
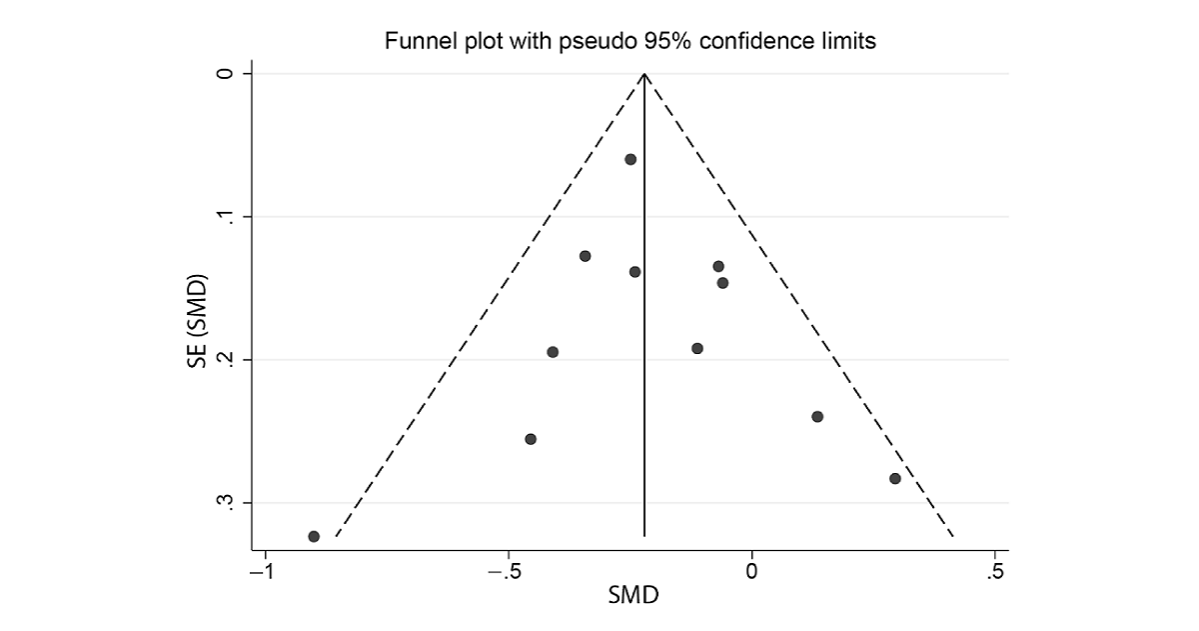
Funnel plot for anxiety meta-analysis. SMD: standardized mean difference.

A total of 11 studies were included in the anxiety meta-analysis (3/11, 27% good quality; 2/11, 18% fair quality; and 6/11, 55% poor quality) [39-42,44,45,48,49,52-54]. The pooled SMD for the anxiety random-effects meta-analysis demonstrated a small but statistically significant effect of cCBT, with treatment arms showing lower anxiety scores at follow-up than the control arms (SMD −0.21, 95% CI −0.33 to −0.09; Figure 2). There was evidence of moderate, but not statistically significant, heterogeneity (*I^2^*=36.2%; *Q*_10_=15.68; *P=*.11). Visual inspection of the funnel plot (Figure 3) and the Egger test for funnel plot asymmetry (*P=*.80) did not show strong evidence of publication bias.

Sensitivity analyses yielded similar small effect sizes when the meta-analysis was limited to different intervention, randomization, and analysis types (Table 3). However, the pooled effect size varied according to the risk of bias ratings, with those rated *fair* finding the weakest evidence for an effect of cCBT (Figure S1 in Multimedia Appendix 6 [39-42,44,45,48,49,52-54]). The pooled effect size was substantially smaller and not significant when the control groups were limited to those with an active treatment component.

**Table 3:** Results of stratified anxiety meta-analyses.

Stratified analyses			Quality of the included studies	Total studies, n	Standardized mean difference (95% CI)	Cochran *Q* (df)	Cochran *Q*, *P* value	*I*^2^ (%)
**Randomization type**								
		Clustered [42,49,53]	1 good; 2 poor	3	−0.22 (−0.32 to −0.12)	1.52 (2)	.47	0.0
		Other [39-41,44,45,48,52,54]	2 good; 2 fair; 4 poor	8	−0.22 (−0.42 to −0.02)	14.16 (7)	.048	50.6
**Analysis type**								
		Intention to treat [39,44,45,52]	1 good; 2 fair; 1 poor	4	−0.14 (−0.34 to 0.05)	4.10 (3)	.25	26.9
		Other or unclear [40-42,48,49,53,54]	2 good; 5 poor	7	−0.25 (−0.41 to −0.09)	10.84 (6)	.09	44.7
**Intervention type**								
		Treatment [39-42,45,48,49,52,54]	2 good; 2 fair; 5 poor	9	−0.18 (−0.33 to −0.03)	14.60 (8)	.07	45.2
		Prevention [44,53]	1 good; 1 poor	2	−0.30 (−0.48 to −0.11)	0.30 (1)	.59	0.0
**Control group type**								
	Treatment [39,45,49]		3 good; 1 fair; 5 poor	3	−0.07 (−0.26 to 0.12)	0.78 (2)	.68	0.0
	Other [39-42,44,48,49,52-54]		1 good; 2 fair	10	−0.23 (−0.36 to 0.10)	14.31 (9)	.11	37.1
**Study quality**								
		Good [40,44,49]	3 good	3	−0.25 (−0.47 to −0.03)	2.98 (2)	.23	32.8
		Fair [39,45]	2 fair	2	−0.08 (−0.31 to 0.15)	0.05 (1)	.83	0.0
		Poor [41,42,48,52-54]	6 poor	6	−0.22 (−0.43 to −0.02)	10.97 (5)	.05	54.4

### Depression Meta-analysis

**Figure 4:**
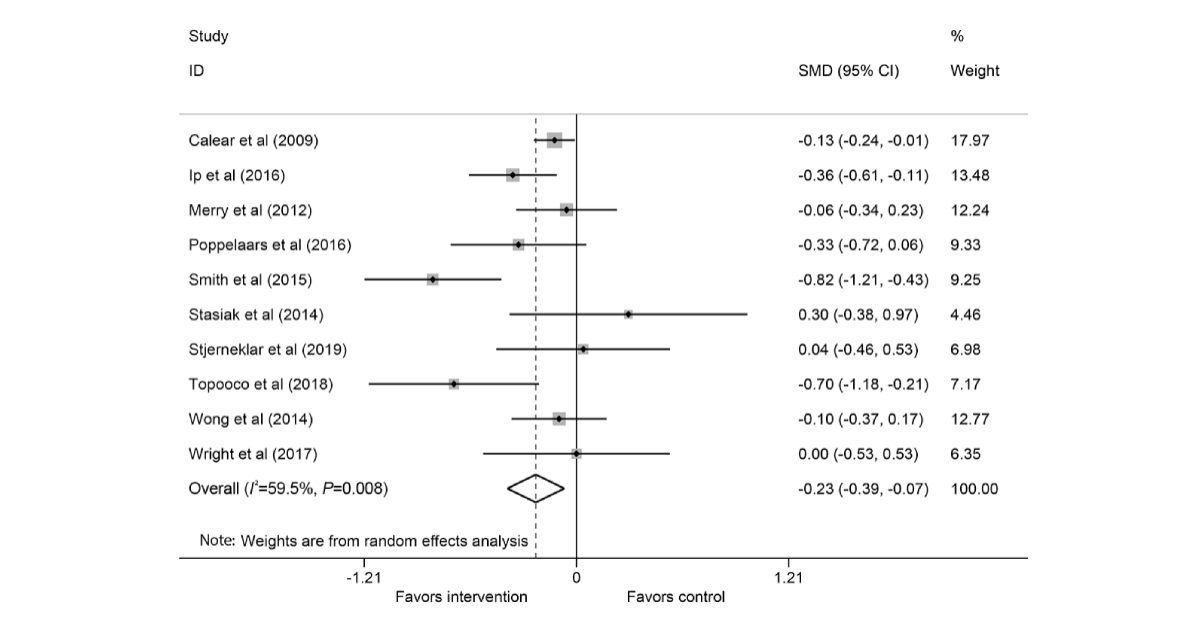
Forest plot for depression meta-analysis. SMD: standardized mean difference.

**Figure 5:**
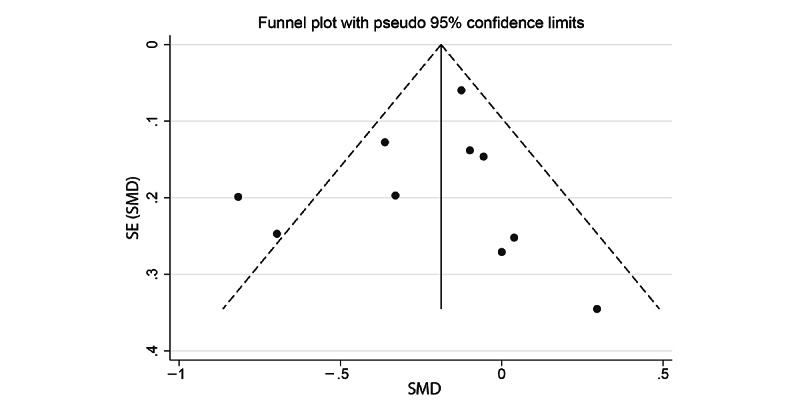
Funnel plot for depression meta-analysis. SMD: standardized mean difference.

A total of 10 studies were included in the depression meta-analysis (3/10, 30% good; 1/10, 10% fair; and 6/10, 60% poor) [40,42,44-46,48,51-54]. As with the anxiety meta-analysis, the pooled SMD for the depression random-effects meta-analysis demonstrated a small but statistically significant effect of cCBT, with treatment arms showing lower depression scores at follow-up than the control arms (SMD −0.23; 95% CI −0.39 to −0.07; Figure 4). There was evidence of moderate and statistically significant heterogeneity (*I^2^*=59.5%; *Q*_9_=22.2; *P=*.008). Visual inspection of the funnel plot (Figure 5) and the Egger test for funnel plot asymmetry (*P=*.53) did not show strong evidence of publication bias.

Sensitivity analyses yielded similar small effect sizes when the meta-analysis was limited to different intervention types, randomization types, and analysis types (Table 4). The effect size again varied according to the risk of bias ratings, with those rated *poor* finding the strongest evidence for an effect of cCBT compared with control arms (note that only one study was rated *fair* in this meta-analysis; Figure S2 in Multimedia Appendix 7 [40,42,44-46,48,51-54]). Finally, as with anxiety, the effect size was substantially smaller and not significant when the control groups were limited to those with an active treatment component.

**Table 4:** Results of stratified depression meta-analyses.

Stratified analyses							Quality of the included studies		Total number of studies, N		Standardized mean difference (95% CI)		Cochran *Q* (df)		Cochran *Q*, *P* value		*I*^2^ (%)
**Randomization type**																	
	Clustered [42,46,53]					3 poor		3		−0.14 (−0.24 to −0.03)		1.07 (2)		.59		0.0	
	Other [40,44,45,48,51,52,54]					3 good; 1 fair; 3 poor		7		−0.26 (−0.53 to 0.01)		18.32 (6)		.005		67.2	
**Analysis type**																	
		Intention to treat [44,45,51,52]				2 good; 1 fair; 1 poor		4		−0.24 (−0.56 to −0.07)		8.29 (3)		.04		63.8	
		Other or unclear [40,42,46,48,53,54]				1 good; 5 poor		6		−0.22 (−0.43 to −0.01)		13.14 (5)		.02		62.0	
**Intervention type**																	
			Treatment [40,42,45,48,51,52,54]			2 good; 1 fair; 4 poor		7		−0.22 (−0.46 to 0.03)		19.18 (6)		.004		68.7	
			Prevention [44,46,53]			1 good; 2 poor		3		−0.26 (−0.43 to −0.09)		2.12 (2)		.35		5.9	
**Control group type**																	
				Treatment [45,46]		1 fair; 1 poor		2		−0.17 (−0.49 to 0.14)		1.47 (1)		.23		32.1	
				Other [40,42,44,46,48,51-54]		3 good; 6 poor		9		−0.25 (−0.43 to −0.07)		21.01 (8)		.007		61.9	
**Study quality**																	
					Good [40,44,51]	3 good		3		−0.10 (−0.49 to 0.29)		4.52 (2)		.11		55.7	
					Fair [45]	1 fair		1		−0.06 (−0.34 to 0.23)		N/A^a^		N/A		N/A	
					Poor [42,46,48,52-54]	6 poor		6		−0.32 (−0.56 to −0.08)		16.74 (5)		.005		70.1	

^a^N/A: not applicable. Only 1 study was included in this stratification.

## Discussion

### Principal Findings

This review aims to provide an update to previous meta-analyses and evaluate the efficacy of cCBT in the treatment of anxiety and depression in adolescents. We found evidence of small but significant effects of cCBT on adolescent anxiety and depression. Secondary analyses suggested that the efficacy of cCBT was comparable with that of alternative, active interventions (such as face-to-face therapy or treatment as usual) and resulted in significantly greater symptom reductions than the control groups not receiving such interventions. However, it should also be noted that we identified a large number of poor-quality studies, which could limit the strength of our overall findings, and it would be important for future RCTs in this area to adopt rigorous methodologies to ensure their reliability and validity.

To the best of our knowledge, this is the first meta-analysis of this kind to focus exclusively on the adolescent age range to establish the efficacy of cCBT within this distinct developmental period. This is an important addition to the literature, given the unique biological and social transitions associated with adolescence, as well as the associated prevalence rates of mental health disorders in this age group [24-26]. Limiting the included studies to RCTs further strengthens the evidence for the efficacy of cCBT [55]. Our findings, therefore, support the results of a previous review which found favorable effects of cCBT for the treatment of anxiety and depression in adolescents [18] and support a recent move by the NICE guidelines to recommend the use of digital CBT for children and young people with mild depression [7]. Our findings are also similar to those seen in the adult population; for example, a recent meta-analysis found that internet-based CBT or cCBT was effective for treating depression and anxiety among adults and showed equivalent effects to face-to-face therapy [56]. Furthermore, we identified studies from a wide range of countries, suggesting that cCBT might be an effective intervention in various cultural settings. However, we did not identify any studies published in languages other than English or conducted in low- and middle-income countries, where a lack of resources and mental health services might make the availability of cCBT particularly beneficial.

Challenges remain in the development of effective digital interventions for adolescents; however, our findings suggest that cCBT may overcome various barriers to treatment in the adolescent age group [14,15] and provide reasonable grounds for optimism in promoting adolescent public health. It was found that cCBT may be a less resource-intensive alternative to traditional therapies, with many programs included in this meta-analysis requiring little or no clinician support. However, receiving professional support alongside computerized therapies might be beneficial, such that blended approaches could be optimal [57]. In addition, parental involvement in child CBT may lead to better outcomes, although evidence on their role in adolescent CBT is less conclusive [58,59]. Few of the studies we identified described direct parental involvement in the cCBT intervention; for those that did, the extent of parental involvement was minimal and sometimes optional. Therefore, the potential role of therapists and parents in adolescent cCBT is an important area for future work to optimize the balance of effectiveness, resource requirements, and scalability. It should also be noted that only 6% (1/16) of the included studies included a health economic analysis; a thorough investigation of the cost-effectiveness of cCBT interventions would be an important area for future studies [54].

The feasibility and acceptability of cCBT are not universally positive, necessitating a thorough understanding of the active components of these interventions [14]. Indeed, this issue is not unique to digital interventions targeting anxiety and depression or to adolescents. Going forward, more studies should demonstrate an awareness of the importance of co-design, personalization, and data privacy, and should incorporate principles from various disciplines such as app design and machine learning to further improve the feasibility and acceptability of these interventions [60-62]. Although this meta-analysis did not explore the quality or usability of interventions, future authors assessing cCBT should also consider using process evaluation measures [63], as has been previously highlighted [64]. This would also help prepare interventions for adoption in schools or routine clinical care, as several of the interventions identified in this study are not currently being implemented outside of a research setting.

### Limitations

This study had several limitations. In the meta-analysis, we were unable to correct for the effects of clustered randomization because of underreporting in many of the included clustered RCTs. Therefore, the effects of clustering were explored using sensitivity analyses, and effect sizes were found to be similar following the removal of clustered RCTs. In addition, the results from the longest available follow-up period were used in the meta-analysis to ensure that prolonged intervention effects could be captured. However, because the longest follow-up period varies among studies, this can introduce additional heterogeneity (Cochrane Handbook section 9.3.4 [31]). Heterogeneity was generally moderate to high, suggesting that some methodological variation among the included studies was not accounted for. Further heterogeneity may have been introduced by combining prevention and treatment studies and by combining superiority and noninferiority studies; nonetheless, we conducted sensitivity analyses to understand the differential effects of these study designs.

We focused on participants aged 11 to 19 years but acknowledge that the age of adolescence is widely debated, such that some of the studies excluded from this review owing to the age of their participants might have produced findings relevant to adolescent groups [24]. The authors of included studies were not contacted to identify further studies for inclusion; however, our search strategy was otherwise rigorous. Finally, the strength of our findings is necessarily limited by the quality of the included studies, which were very mixed, with more than half (9/16, 56%) receiving overall ratings of poor study quality according to our quality assessments [32,33]. Indeed, evidence for the efficacy of cCBT in treating depression was stronger in studies rated as poor quality.

### Conclusions

This meta-analysis reinforces cCBT as an effective intervention for anxiety and depression, showing small, but statistically significant effects. To our knowledge, this is the first study to establish this relationship in an exclusively adolescent group. Most studies were of poor quality and highly heterogeneous, highlighting the need for rigorous and high-quality RCTs in this area. Given the wide variety of available programs and technologies, future research could focus on establishing the active components of cCBT and draw principles from various disciplines, such as design technology and computer science, to optimize feasibility and acceptability. Nonetheless, the clinical potential of cCBT in treating adolescent anxiety and depression is clear and has the scope to address current unmet needs within child and adolescent mental health services.

## Appendix

Multimedia Appendix 1 PRISMA (Preferred Reporting Items for Systematic Reviews and Meta-Analyses) checklist.

Multimedia Appendix 2 Search strategy.

Multimedia Appendix 3 Included studies’ populations and exclusion critieria.

Multimedia Appendix 4 Intervention descriptions.

Multimedia Appendix 5 Data used in meta-analyses.

Multimedia Appendix 6 Anxiety forest plot by study quality.

Multimedia Appendix 7 Depression forest plot by study quality.

## Abbreviations

CBT: cognitive behavioral therapy
cCBT: computerized CBT
NHS: National Health Service
NICE: National Institute for Health and Care Excellence
NIHR: National Institute for Health Research
PRISMA: Preferred Reporting Items for Systematic reviews and Meta-Analyses
RCT: randomized controlled trial
SMD: standardized mean difference
