# Supplementary material for: Computerized Cognitive Behavioral Therapy for Treatment of Depression and Anxiety in Adolescents: Systematic Review and Meta-analysis
Source: J Med Internet Res. 2022 Apr 11;24(4):e29842. doi: 10.2196/29842 (PMC9039813; doi:10.2196/29842)
Supplement: Multimedia Appendix 2 [file jmir_v24i4e29842_app2.docx]

*TableS2: Search strategy*

|  | **Searches** | **Results** |
| --- | --- | --- |
| **Database 1: Ovid Medline ® -** And In-Process & Other Non-Indexed Citations – 1946 to July 1^st^ 2019 | | |
| #1 | iCBT OR CCBT OR “Computer-based CBT” OR “Computer assisted CBT” OR “Mobile CBT” OR “Digital CBT” OR “Internet CBT”.mp | 740 |
| #2 | Adolescen* OR teen* OR child*.mp | 3,257,554 |
| #3 | Depress* OR anx*.mp | 614,355 |
| #4 | 1 and 2 and 3 | 126 |
|  |  |  |
| **Database 2: Embase –** 1974 to 2019 Week 26 | | |
| #1 | iCBT OR CCBT OR “Computer-based CBT” OR “Computer assisted CBT” OR “Mobile CBT” OR “Digital CBT” OR “Internet CBT”.mp | 1040 |
| #2 | Adolescen* OR teen* OR child*.mp | 3,106,220 |
| #3 | Depress* OR anx*.mp | 885,086 |
| #4 | 1 and 2 and 3 | 108 |
|  |  |  |
| **Database 3: PsychInfo –** 1806 to July Week 1 2019 | | |
| #1 | iCBT OR CCBT OR “Computer-based CBT” OR “Computer assisted CBT” OR “Mobile CBT” OR “Digital CBT” OR “Internet CBT”.mp | 541 |
| #2 | Adolescen* OR teen* OR child*.mp | 845,748 |
| #3 | Depress* OR anx*.mp | 454,270 |
| #4 | 1 and 2 and 3 | 82 |
|  |  |  |
|  | **Total Articles (Before Removing Duplicates):** | **316** |
