# Supplementary material for: Computerized Cognitive Behavioral Therapy for Treatment of Depression and Anxiety in Adolescents: Systematic Review and Meta-analysis
Source: J Med Internet Res. 2022 Apr 11;24(4):e29842. doi: 10.2196/29842 (PMC9039813; doi:10.2196/29842)
Supplement: Multimedia Appendix 3 [file jmir_v24i4e29842_app3.docx]

*TableS3: Included studies’ populations and exclusion criteria*

| **Study** | **Population** | **Participant exclusion criteria** |
| --- | --- | --- |
| Calear et al., 2009 | Students from Australian schools | None |
| Fleming et al., 2012 | Adolescents excluded from mainstream education | Severe depression; High suicide risk |
| Ip et al., 2016 | Adolescents from Hong Kong secondary schools | CESD-R score >40 and <12; Diagnosed with MDD, Schizophrenia or Bipolar; Hospitalization risk due to suicidal attempts; Antidepressant/Psychotropic use; Substance abuse; Reading impairment; Intellectual disability; Visual impairment; Developmental disability |
| Merry et al., 2012 | Adolescents diagnosed with mild to moderate depressive disorder by a clinician | Depression too severe to make a self-help resource a viable option; Adolescent at high risk of self-harm or suicide; Scored 7 on item 12 (morbid ideation) or ≥5 on item 13 (suicidal ideation) on CDRS-R; Scored <30 on CDRS-R; Intellectual disability or physical limitations precluding use of computer program; Major mental health disorder where primary focus not depression; Received CBT, interpersonal therapy or antidepressants in preceding 3 months. |
| Poppelaars et al., 2016 | Dutch adolescent girls with subclinical depressive symptoms | Suicidal ideation at screening; Currently receiving mental health care. |
| Sekizaki et al., 2019 | Young male athletes from a school in Japan | None |
| Smith et al., 2015 | Adolescents in the UK | Severe symptoms and/or presence of significant risk requiring immediate attention |
| Spence et al., 2011 | Adolescents in Australia suffering from GAD, SoP, SAD, SP | Primary diagnosis of PD, OCD, PTSD; Mood disturbance rated “moderately disturbing” or greater (severity rating of 6 or higher on ADIS-C); PDD; Learning disorder; Behavioural disorder; Substance abuse; Suicidal ideation; Self-harm |
| Sportel et al., 2013 | Adolescents in the Netherlands with social anxiety and/or test anxiety | DSM-IV diagnoses other than anxiety; Severe anxiety; Receiving regular treatment |
| Stallard et al., 2011 | Adolescents diagnosed with a primary anxiety disorder or mild to moderate depression as diagnosed by a clinician | English not first language; Severe depression; Serious self-harm; Psychosis; Recent abuse victim; PTSD; autism; ADHD; Learning difﬁculties |
| Stasiak et al., 2014 | Adolescents that self-referred to a school counsellor for help with low mood | Moderate/high suicide risk; Receiving psychological therapy; Moderate/severe learning disability; Limited English language; No computer access |
| Stjerneklar et al., 2019 | Adolescents that self-referred to a psychological research and testing facility in Denmark | Severe comorbid depression (DSM-IV ADIS-C/P >5); substance abuse; current severe self-harm or suicidal ideation; PDD; Learning disorder or intellectual disability; Psychotic symptoms |
| Topooco et al., 2018 | Adolescents that volunteered to the study, fulfilling criteria for depression or MDD as diagnosed by a clinician | Severe suicidal ideation (MINI ≤16); Suicidal ideation (PHQ-9≤1); Comorbid psychiatric conditions; Receiving psychological treatment; Substance misuse (MINI) |
| Wong et al., 2014 | Year 9-10 students from Australian Secondary schools | None |
| Wright et al., 2017 | Adolescents referred by Primary Mental Health Workers in CAMHS centres for adolescents diagnosed with low mood/depression | Psychosis, active suicidality or postnatal depression; Those with severe depression were referred to CAMHS for assessment and treatment, then clinicians decided whether trial participation would be appropriate. |
| Wuthrich et al., 2012 | Adolescents who met DSM-IV criteria for an anxiety disorder as diagnosed by a clinician (Disorders included SAD, GAD, & Social Phobia) | Self harm; Suicidal ideation; Psychosis; Bipolar Disorder; Sexual/Physical abuse; More than one grade behind peers |

Abbreviations: ADHD (Attention Deficit Hyperactivity Disorder); ADIS-C/P (Anxiety Disorders Interview Schedule – Child/Parent version); CAMHS (Child and Adolescent Mental Health Services); CDRS-R (Children’s Depression Rating Scale-Revised); CESD-R (revised Center for Epidemiologic Studies Depression Scale); DSM-IV (Diagnostic and Statistical Manual of Mental Disorders, 4th Edition); GAD (Generalized Anxiety Disorder); MDD (Major Depressive Disorder); MINI (Mini-International Neuropsychiatric Interview); OCD (Obsessive Compulsive Disorder); PD (Panic Disorder); PDD (Pervasive Developmental Disorder); PHQ-9 (Patient Health Questionnaire); PTSD (Posttraumatic Stress Disorder); SAD (Separation Anxiety Disorder); SoP (Social Phobia); SP (Specific Phobia).
