# Supplementary material for: Computerized Cognitive Behavioral Therapy for Treatment of Depression and Anxiety in Adolescents: Systematic Review and Meta-analysis
Source: J Med Internet Res. 2022 Apr 11;24(4):e29842. doi: 10.2196/29842 (PMC9039813; doi:10.2196/29842)
Supplement: Multimedia Appendix 4 [file jmir_v24i4e29842_app4.docx]

*TableS4: Intervention descriptions*

| **Intervention** | **Description** | **Extent of therapist or clinician involvement** | **Extent of parent involvement** | **To the corresponding author’s knowledge, is the intervention available or being delivered outside of the research setting?** |
| --- | --- | --- | --- | --- |
| BRAVE-Online | The intervention is based on theoretical and empirical research related to the psychosocial determinants of anxiety. Targets four types of anxiety: Generalized Anxiety Disorder, Social Phobia, Separation Anxiety Disorder, and Specific Phobia. Content and session length replicates face to face CBT therapy and includes psychoeducation, relaxation training, recognition of the physiological symptoms of anxiety, coping strategies, cognitive restructuring, and graded exposure. | Spence et al. (2011): 15 minute phone call with a therapist following session 5, therapists also monitor and provide feedback on progress. | Spence et al. (2011): Parents received 5 sessions (plus 2 booster sessions) on acquiring CBT techniques, and parenting strategies to help the adolescent use skills to handle anxious situations. | Spence et al. (2011): BRAVE has been disseminated nationally in Australia. It is available in all three forms (child, adolescent and pre-school) to all Australian families free of charge. |
| ChilledOut online | Danish version of ChilledOut online, a web-based intervention adapted from the Cool Kids child anxiety management program^[[1]](#endnote-1)^. Based on CBT principles, users work through 8 modules approximately 30 minutes long. The intervention focuses on psychoeducation, cognitive restructuring and graded exposure. The version used was an adapted Danish version. | Stjerneklar et al. (2019): Email and therapist support offered through the intervention. | Stjerneklar et al. (2019): Parents received the ChilledOut Parent Companion explaining the treatment and advising on how to support the adolescent. Parent and therapist telephone contact. Parents could also reach out to the therapist, and join an online group network. | Stjerneklar et al. (2019): No. |
| Cool Teens | CD-Rom intervention based on the Cool Kids anxiety management CBT program. There are eight modules of 30 minutes each, with a strong focus on cognitive restructuring and graded exposure. Combination of multimedia formats are used (cartoons, illustrations, videos, text, audio) to create engaging content, activities, and homework assignments. | Wuthrich et al. (2012): Therapists support participants through phone calls to problem solve on 8 occasions throughout the intervention. | Wuthrich et al. (2012): Adolescents were supported by their parent to an extent decided by the adolescent. Parents received handouts on the core strategies so that they could support the adolescent. Parent and therapist telephone contact. | Wuthrich et al. (2012): The intervention has been moved to an internet version and renamed ChilledOut^i^. It is being used in various settings such as in health clinics and schools nationally in Australia (as well as internationally in research trials). |
| Grasp The Opportunity | Grasp the opportunity is a web-based CBT intervention designed for Chinese adolescents with mild depression. It is a modified version of CATCH-IT (Competent Adulthood Transition with Cognitive Behavioural Humanistic and Interpersonal Training) which incorporates CBT, behavioural activation, and interpersonal psychotherapy. | Ip et al. (2016): Therapist intervention offered if participants were in crisis, otherwise unsupported. | Ip et al. (2016): No involvement reported. | Ip et al. (2016): No information given. |
| MoodGYM | MoodGYM is a web-based CBT program designed to target adolescent anxiety and depression. Its fundamental goal is to change dysfunctional thoughts, improve self-esteem and interpersonal relationships, and teach important life skills, for example problem solving and relaxation. Users work through five interactive modules containing information, animated demonstrations, quizzes, and homework. | Calear et al. (2009): No involvement reported. | Calear et al. (2009): No involvement reported. | Calear et al. (2009): MoodGYM can be accessed by the public as a self-help program. It is available free of charge in Australia and may have a small fee for users outside of Australia. MoodGYM is used in schools and in the community, as an adjunct to clinical services. |
| SPARX | Interactive game consisting of psychoeducation, relaxation skills, problem solving, activity scheduling, challenging and replacing negative thinking and social skills. The programme includes direct instructional content as well as narrative and experiential learning components. Each module combines use of a "guide" that teaches and instructs, as well as a game component where users inhabit a personalised character and complete missions. | Fleming et al. (2012): No involvement reported.  Merry et al. (2012): Young people who were not improving were prompted to seek help from their referring clinicians.  Poppelaars et al. (2016): No involvement reported. | Fleming et al. (2012): No involvement reported.  Merry et al. (2012): No involvement reported.  Poppelaars et al. (2016): No involvement reported. | Fleming et al. (2012): SPARX is publicly available for free as a self-help tool with no referral needed to people with a NZ IP address. There is also a Japanese version which is available for purchase on the Japanese app store.  Merry et al. (2012): SPARX was launched as a national programme in New Zealand in April 2014 under funding from the government. It is freely available on the internet and as an app. It is often recommended by school personnel and by health care practitioners but access is not controlled by them. It is also available commercially in Japan.  Poppelaars et al. (2016): No information given. |
| Stressbusters | Based on effective face to face CBT protocol for adolescents with depression. The programme includes: psychoeducation, behavioural activation, identifying and changing negative automatic thoughts, improving problem solving, improving social skills, and relapse prevention | Smith et al. (2015): No involvement reported.  Wright et al. (2017): No involvement reported. | Smith et al. (2015): No involvement reported.  Wright et al. (2017): No involvement reported. | Smith et al. (2015): No.  Wright et al. (2017): No information given. |
| The Journey | CD-ROM based CBT embedded in a fantasy game-like environment incorporating animation, text, sound, videos, and interactive exercises. The user follows a narrative quest linked to a cCBT technique (for example, cognitive restructuring), and earn points for completing modules. The Journey includes seven modules, which take approximately 25–30 minutes to complete. | Stasiak et al. (2014): No involvement reported. | Stasiak et al. (2014): No involvement reported. | Stasiak et al. (2014): No information given. |
| Think, Feel, Do | Six session CD-ROM based on CBT workbook created by Stallard. Facilitated by a helper (psychology assistant, teacher, or nurse) that elaborates on the CD-ROM content as well as provides support and clarifies misunderstandings. Uses sounds, photos, music, cartoons, and quizzes to guide the user through the sessions and is made to be very interactive. | Stallard et al. (2011): Sessions facilitated by a professional such as a psychology assistant, teacher or nurse. | Stallard et al. (2011): No involvement reported. | Stallard et al. (2011): No information given. |
| ThisWayUp | Web-based intervention targeting Depression and Anxiety. ThisWayUp was designed as universal prevention and is based on CBT principles. The Depression element contains 8 sessions, and the anxiety 7, with each session lasting 40 minutes. The programmes incorporate CBT components based on skill acquisition including: psychoeducation, management of thoughts, emotions, and behaviours, and is facilitated by a classroom teacher. | Wong et al. (2014): No involvement reported. | Wong et al. (2014): No involvement reported. | Wong et al. (2014): Courses described have been more recently examined by Teesson et al. (2020)^[[2]](#endnote-2)^. Teesson et al. report that the courses were extensively modified, and are available for schools in the trial through Climate Schools. |
| Unnamed intervention (Sekizaki et al., 2019) | A highly structured web-based CBT program based on effective face to face CBT protocol. The intervention included eight skills-based modules and weekly therapist chat sessions lasting approximately 30 minutes. Modules targeted behavioural and cognitive factors associated with anxiety and depression through a number of techniques including psychoeducation, behavioural activation, cognitive restructuring, affect regulation, anxiety management, and relapse prevention. Users worked through reading material, videos, fictional patient stories, interactive tasks and homework. | Sekizaki et al. (2019): cCBT delivered alongside nurse lead CBT education sessions. | Sekizaki et al. (2019): No involvement reported. | Sekizaki et al. (2019): Available in schools or in the workplace. Services delivered in schools as part of the curriculum to improve student’s mental health. |
| Unnamed intervention (Sportel et al., 2013) | This web-based intervention contained 20, 40 minute sessions delivered twice a week. Based on the principles of CBT, the intervention aimed to target attentional bias, interpretive bias, dysfunctional associations, and implicit self esteem in socially anxious adolescents using Cognitive Bias Modification. Users work through a number of tasks and scenarios including word sorting and visualisation activities. | Sportel et al. (2013): No involvement reported. | Sportel et al. (2013): No involvement reported. | Sportel et al. (2013): No. |
| Unnamed intervention (Topooco et al., 2018) | A highly structured web-based CBT program based on effective face to face CBT protocol and suitable for use on computers, smartphones and tablets. The intervention included eight skills-based modules and weekly therapist chat sessions lasting approximately 30 minutes. Modules targeted behavioural and cognitive factors associated with anxiety and depression through a number of techniques including psychoeducation, behavioural activation, cognitive restructuring, affect regulation, anxiety management, and relapse prevention. Users worked through reading material, videos, fictional patient stories, interactive tasks and homework. | Topooco et al. (2018): Weekly therapist chat session. | Topooco et al. (2018): No involvement reported. | Topooco et al. (2018): No. |

1. Lyneham HJ, McLellan LF, Cunningham M, Wuthrich V, Schniering C, Hudson JL, et al. ChilledOut Online Program. Sydney: Centre for Emotional Health, Macquarie University; 2014. [↑](#endnote-ref-1)
2. Teesson, M., Newton, N. C., Slade, T., Chapman, C., Birrell, L., Mewton, L., ... & Andrews, G. (2020). Combined prevention for substance use, depression, and anxiety in adolescence: a cluster-randomised controlled trial of a digital online intervention. The Lancet Digital Health, 2(2), e74-e84. [↑](#endnote-ref-2)
