# Supplementary material for: Computerized Cognitive Behavioral Therapy for Treatment of Depression and Anxiety in Adolescents: Systematic Review and Meta-analysis
Source: J Med Internet Res. 2022 Apr 11;24(4):e29842. doi: 10.2196/29842 (PMC9039813; doi:10.2196/29842)
Supplement: Multimedia Appendix 5 [file jmir_v24i4e29842_app5.docx]

*TableS5: Data used in meta-analyses*

| **Lead author, year** | **Intervention type (treatment/ prevention)** | **Randomisation type (clustered/**  **other)** | **Analysis type (ITT/other or unclear)** | **Control type (treatment/**  **other)** | **Measure used in meta-analysis** | **Intervention arm(s): follow-up n** | **Intervention arm(s): follow-up M** | **Intervention arm(s): follow-up SD** | **Control arm(s): follow-up n** | **Control arm(s): follow-up M** | **Control arm(s): follow-up SD** |
| --- | --- | --- | --- | --- | --- | --- | --- | --- | --- | --- | --- |
| **ANXIETY** | | | | | | | | | | | |
| Calear,  2009 | Treatment | Clustered | Other or unclear | Other | RCMAS | 455 | 6.98 | 6.34 | 734 | 8.65 | 6.9 |
| Ip,  2016 | Prevention | Other | ITT | Other | DASS | 123 | 5.16 | 3.26 | 127 | 6.39 | 3.86 |
| Merry,  2012 | Treatment | Other | ITT | Treatment | SCAS | 94 | 25.6 | 10.32 | 93 | 26.25 | 11.28 |
| Smith,  2015 | Treatment | Other | Other or unclear | Other | SCARED | 53 | 23.8 | 18.4 | 55 | 31.2 | 17.5 |
| Spence,  2011 | Treatment | Other | ITT | Arm 1: Other  Arm 2: Treatment | SCAS-C | 44 | 27.78 | 15.99 * | Arm 1: 27  Arm 2: 44 | Arm 1: 26.52  Arm 2: 31.54 | Arm 1: 16.52 *  Arm 2: 16.65 * |
| Sportel,  2013 | Treatment | Clustered | Other or unclear | Arm 1: Treatment  Arm 2: Other | RCADS social phobia | 86 | 10.15 | 5.73 | Arm 1: 84  Arm 2: 70 | Arm 1: 10.13  Arm 2: 10.94 | Arm 1: 4.7  Arm 2: 4.55 |
| Stjerneklar,  2019 | Treatment | Other | Other or unclear | Other | SCAS-C | 32 | 31.88 | 16.06 | 31 | 40.19 | 19.9 |
| Topooco,  2018 | Treatment | Other | ITT | Other | BAI | 33 | 20.6 | 9 | 37 | 19.4 | 8.6 |
| Wong,  2014 | Prevention | Clustered | Other or unclear | Other | GAD-7 | Arm 1: 101  Arm 2: 92 | Arm 1: 3.47  Arm 2: 3.83 | Arm 1: 3.19  Arm 2: 3.17 | 72 | 4.41 | 3.22 |
| Wright,  2017 | Treatment | Other | Other or unclear | Other | SCAS | 23 | 46.7 | 23 | 28 | 39.9 | 22.5 |
| Wuthrich,  2012 | Treatment | Other | Other or unclear | Other | SCAS-P | 24 | 23 | 19.94 * | 19 | 40.72 | 18.48 * |
| **DEPRESSION** | | | | | | | | | | | |
| Calear,  2009 | Treatment | Clustered | Other or unclear | Other | CESD | 454 | 10.56 | 10.01 | 735 | 11.92 | 11.36 |
| Ip,  2016 | Prevention | Other | ITT | Other | CESD-R | 123 | 20.59 | 8.41 | 127 | 23.66 | 8.5 |
| Merry,  2012 | Treatment | Other | ITT | Treatment | RADS-2 | 94 | 60.68 | 12.51 | 93 | 61.45 | 14.68 |
| Poppelaars,  2016 | Prevention | Clustered | Other or unclear | Arm 1: Other  Arm 2: Treatment | RADS-2 | 38 | 57.08 | 14.21 | Arm 1: 47  Arm 2: 36 | Arm 1: 61.22  Arm 2: 62.44 | Arm 1: 15.03  Arm 2: 12.77 |
| Smith,  2015 | Treatment | Other | Other or unclear | Other | MFQ-C | 55 | 13.4 | 12.9 | 55 | 24.3 | 13.6 |
| Stasiak,  2014 | Treatment | Other | ITT | Other | RADS-2 | 17 | 62.35 | 13.03 | 17 | 58.24 | 14.14 |
| Stjerneklar,  2019 | Treatment | Other | Other or unclear | Other | S-MFQ | 32 | 8.06 | 7.77 | 31 | 7.77 | 7.14 |
| Topooco,  2018 | Treatment | Other | ITT | Other | BDI-II | 33 | 19.9 | 7.2 | 37 | 25.2 | 7.8 |
| Wong,  2014 | Prevention | Clustered | Other or unclear | Other | PHQ-5 | Arm 1: 101  Arm 2: 92 | Arm 1: 2.32  Arm 2: 2.55 | Arm 1: 2.42  Arm 2: 2.41 | 72 | 2.67 | 2.47 |
| Wright,  2017 | Treatment | Other | Other or unclear | Other | S-BDI | 25 | 15.5 | 9.6 | 30 | 15.5 | 10.1 |

* Calculated from the standard error

Abbreviations: [S]-BDI [II] ([Short]-Beck Depression Inventory [II]); (BAI (Beck Anxiety Inventory); CESD-[R] (Center for Epidemiologic Studies Depression Scale-[Revised]); DASS (Depression Anxiety Stress Scale); GAD-7 (Generalised Anxiety Disorder seven item scale); M (mean); [S]-MFQ-[C] ([Short]-Mood and Feelings Questionnaire-[Child Report]); n (sample size); PHQ-5 (Patient Health Questionnaire-5); RADS-2 (Reynolds Adolescent Depression Scale-second edition); RCADS (Revised Child Anxiety and Depression Scale); RCMAS (Revised Children’s Manifest Anxiety Scale); SD (standard deviation); SCARED (Screen for Child Anxiety Related Disorders); SCAS-[C/P] (Spence Children’s Anxiety Scale-[Child/Parent])
