# Supplementary figures and images for: Computerized Cognitive Behavioral Therapy for Treatment of Depression and Anxiety in Adolescents: Systematic Review and Meta-analysis
Source: J Med Internet Res. 2022 Apr 11;24(4):e29842. doi: 10.2196/29842 (PMC9039813; doi:10.2196/29842)

*FigS1: Anxiety forest plot by study quality*


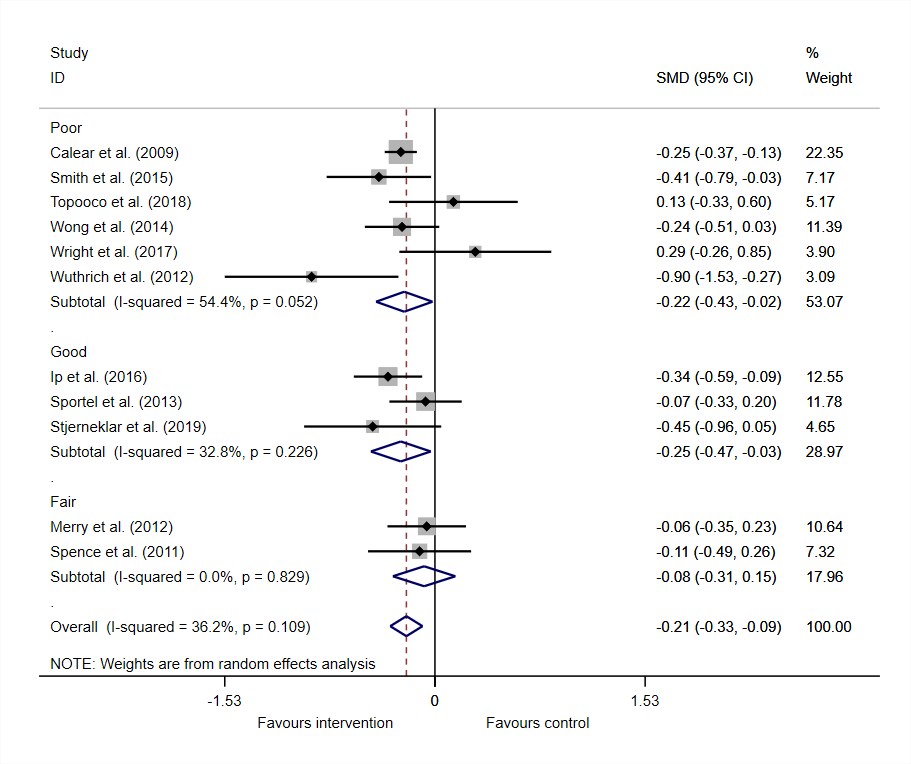

Supplement: Multimedia Appendix 6 [file jmir_v24i4e29842_app6.docx]

*FigS2: Depression forest plot by study quality*

*
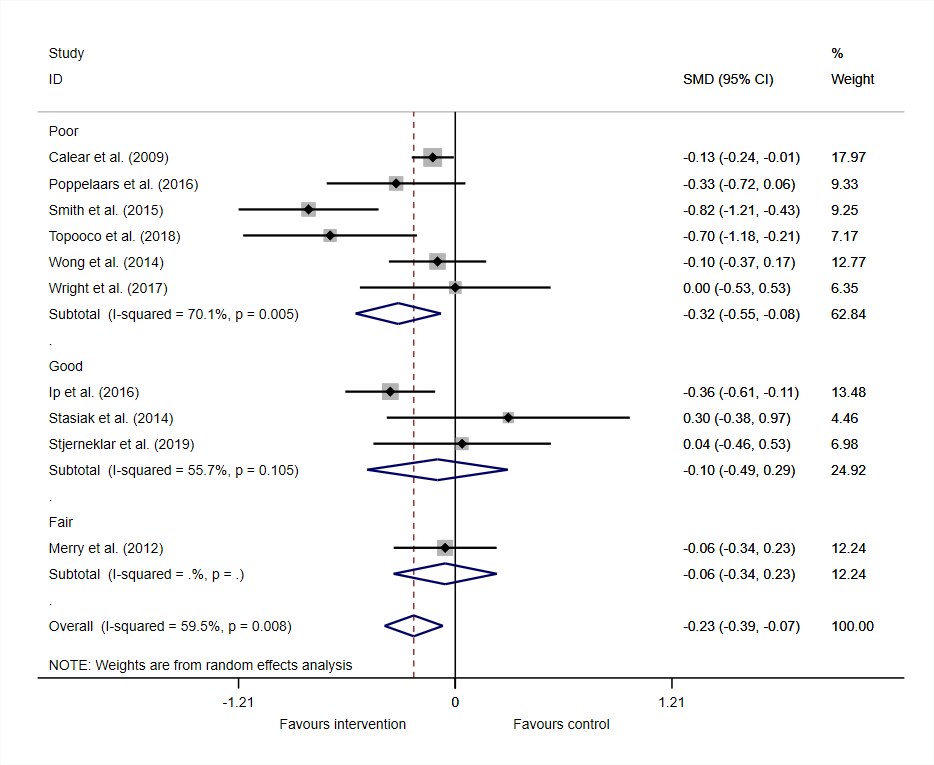
*

Supplement: Multimedia Appendix 7 [file jmir_v24i4e29842_app7.docx]
